# Foodborne Disease and the Need for Greater Foodborne Disease Surveillance in the Caribbean

**DOI:** 10.3390/vetsci4030040

**Published:** 2017-08-11

**Authors:** Brendan Lee

**Affiliations:** School of Veterinary Medicine, Saint Matthews University, Grand Cayman, KY1-1204, Cayman Islands; blee@stmatthews.edu; Tel.: +1-345-945-3199

**Keywords:** foodborne diseases, animals, humans, public health, one health

## Abstract

The Caribbean is a net importer of food, and with billions of dollars’ worth of food products being imported each year, territorial governments are now seeking to encourage local production of foods in an attempt to stem the loss of foreign exchange from these economies with little resilience. The Caribbean, however, lacks the comprehensive food safety system that should be a corollary to successful food production. Regional authorities underestimate the burden of foodborne diseases especially on its workforce and major economic base, the tourism industry. Anecdotally after every mass event in the region, many officially unreported cases of gastroenteritis are recognized. This short communication makes the argument of the importance of food borne illnesses specific to the Caribbean, and improvements that could be made to surveillance to reduce negative outcomes associated with the food supply chain.

## 1. Introduction

The value of healthy food is underscored by the fact it is one of the few commodities that we cannot live without. The World Health Organization (WHO) estimates the annual Global burden of foodborne disease to be over 600,000,000 cases of illness, with almost 420,000 deaths and 27,000,000 Years of Life Lost (YLL) [1]. The sub regional grouping AMR B (Americas Region B), which includes the Caribbean, experienced 140 Disability Adjusted Life Years (DALYs) per 100,000 population, much lower than the African countries at an average of about 1200 (DALYs) per 100,000, but almost four times that experience in Europe (41–52 DALYs), North America and Cuba (35 DALYs) and some of the Asia/Pacific countries (36 DALYs) [1,2]. In the United States the cost of annual foodborne illnesses that affect some 48 million Americans was put at approximately $US78 billion taking into account such factors as cost of health care and loss of productivity [3]; a cost of over $US1600 per person each year. In the Caribbean that burden has been estimated to be approximately $US40 million [4].

The WHO estimates that there are some 31 hazards that cause foodborne illnesses, involving, viruses, bacteria and parasites many of which occur in the Caribbean [5,6,7]. The Caribbean has a culture of purchasing ready to eat foods from street vendors and from vendors at public activities [8] exposing the population to many bacteria that have been associated with foodborne diseases including *Bacillus* spp., *Staphylococcus* spp., *Clostridium* spp., *Vibrio* spp., *Campylobacter* spp., *Listeria* spp., *Salmonella* spp. [9]. Guerra, Almeida, and Willingham [4] identified *Campylobacter* spp., *Escherichia coli* and *Salmonella* spp. as important pathogens found in food animals and products from animal origin in the Caribbean region. Diseases such as toxoplasmosis and viral hepatitis both possibly linked to consumption of contaminated foods have significant prevalence within the Caribbean [10,11].

## 2. The Need for Surveillance

Foodborne disease surveillance is dedicated to assessing the incidence and prevalence of pathogens associated with food and food products with the purpose of providing information to public health authorities that can be used to prevent and control food-related disease outbreaks and also to improve the safety and quality of food products. Such surveillance measures if used appropriately would save lives and reduce the costs associated with foodborne diseases [3,4]. In the Caribbean very little information is available on the morbidity and mortality associated with foodborne diseases or on the attributable sources of the causative pathogens and where they are introduced into the food supply chain. The infectious disease surveillance efforts of the individual regional governments and regional institutions seem to focus episodically on the infectious disease of the day moving from Dengue to Chikungunya to Zika. While these diseases possibly cause greater morbidity and mortality, foodborne diseases should not be overlooked nor should their significant contribution in decreasing the productivity of Caribbean countries. Typically very few samples are submitted for diagnostic testing in Caribbean countries unless an outbreak is detected, most often at a public hospital, which means that many possible cases of disease go undetected through under-reporting and under-diagnosing. The laboratory capacity of the countries of the Caribbean vary with very few of the national health laboratories being accredited [12]. The countries of the Caribbean Community (CARICOM) rely on the Caribbean Public Health Agency (CARPHA) as their reference laboratory for assistance with diagnostic services in identifying foodborne pathogens through sub-typing and other molecular diagnostic techniques as well as the overall surveillance of foodborne pathogens. With limited resources, it is understandable that regional and national surveillance systems will be constrained, but greater effort should be made to involve the private health, agriculture and food production sectors.

Many of the Caribbean islands rely on the tourism industry as a major contributor to the Gross Domestic Product (GDP) of the country; both cruise ship and stay over visitors. The World Travel and Tourism Council [13] estimated that travel and tourism contributed 14.8% or $US53.1 billion to the Caribbean GDP in 2015. Without adequate preventive measures in place, foodborne illnesses could pose a major threat to tourism and the economic output of the region, directly by the exposure of visitors to foodborne pathogens in contaminated foods and workers who do not report their illnesses or indirectly by causing visitor avoidance out of the fear of being exposed to disease pathogens. The resulting loss of market share not only within the tourist industry but also within the local, regional and international food markets would also be devastating to regional economies [14,15]. There is a significant loss of productivity associated with foodborne illness in terms of days of work lost for those suffering from the illness as well as their care-givers [16] which would impact all industries and should be comprehensively evaluated across the region to help quantify the true burden of these diseases in the Caribbean.

The Caribbean has a severe socio-economic gradient with the highest earning 10% earning far more than the lowest 10%. A report by Thomas and Wint [17] identified the ratio comparison of the richest 10% to the poorest 10% in Latin America and the Caribbean at 46 to 1 which is greater than any other region in the world, twice that of Sub-Saharan Africa, and three times than of the industrialized countries. This extreme socioeconomic gradient has been associated with negative health outcomes as has diminished GDP. Unmitigated foodborne disease outbreaks, which potentially can ruin the tourism industry within the Caribbean and decrease productivity in other sectors, could lead to negative health outcomes from reduced capacity of the state to maintain public health and reduced capacity of the individual to afford needed healthcare.

## 3. Preventive Medicine and Foodborne Diseases

The approach to improving food safety and health-related outcomes hinges on a preventive medicine approach rather than reacting to outbreaks and epidemics when they have occurred. This approach would seek to prevent the occurrence of foodborne disease outbreaks or contamination events of the food supply chain. Adopting a One Health Approach, which seeks to draw from cross-disciplinary resources to address health challenges, would provide the best outcome to the complex systems oriented challenges of foodborne disease and food safety [18]. This approach dictates that the stakeholders involved at any level of the food industry from food production to marketing, sales and consumption along with food safety and health must be part of the dialogue on addressing these challenges. The One Health approach if applied properly would engage microbiologists, physicians, veterinarians, public health officials, manufacturers, farmers, sociologists, economists and other disciplines involved in food safety and production. Foodborne disease and food safety is a global challenge and not unique to the Caribbean, with more developed countries investing in molecular diagnostic technologies, syndromic surveillance and extensive electronic platforms to capture, store, analyze and disseminate associated data [19]. Indeed it is the desire of the international foodborne disease surveillance community to move towards whole genome sequencing to provide unambiguous, rapid identification of pathogens improving the preventive and response capabilities of public health agencies [20,21]. While the countries of the Caribbean also are part of this long term vision, lack of infrastructure and necessary professionals make it unrealistic at this time with even the region’s one reference diagnostic center, CARPHA, struggling to meet its regional public health mandate. 

In addition to the use of molecular techniques, many countries have introduced syndromic surveillance, combining information from traditional sources such as hospital patient records with non-traditional sources such as over-the-counter drug sales, animal health data, and environmental data to identify disease outbreaks more quickly than traditional surveillance would allow [22]. Syndromic surveillance also allows for the easier integration of disease modelling and geographic information system (GIS) data. This approach to surveillance while providing the benefit of non-traditional surveillance strategies that may allow quicker recognition and hence control of foodborne disease outbreaks, also carries disadvantages such as the cost of the electronic platforms required to manage this data, the need for compatible infrastructure within collaborating institutions and the need for increased human resources (epidemiologists, data analysts and internet technology experts) [23]. The biggest limitations of syndromic surveillance are the production of vast volumes of data, sometimes of questionable benefit, that such surveillance generates and the systems inability to reliably recognize individual cases [24,25]. This may make implementation of such a system cost prohibitive to the region, though governments should be encouraged to begin integrating surveillance data across disciplines and the real-world application of the resulting analysis where possible and within budgetary constraints.

Working with the national and regional medical and nursing associations is important to remind health practitioners of the value of submitting samples for testing and data collection. This is especially important in the private sector where many cases of possible foodborne diseases may be seen and treated without the submission of samples for diagnostic workup. Hennessey et al. [26] suggested that many physicians would benefit from education on diagnostic fecal tests since familiarity with the pathogens that could be identified and by what tests was at times limited. Undoubtedly as a new technology is developed for diagnostic microbiology, health practitioners need continuing education to keep abreast with the changes. The study by Hennessey et al. [26] also highlighted that physicians were also reluctant to submit samples for testing because it was unlikely to change the recommended treatment protocol. While this may be the case in some situations the value of sample testing is heavily epidemiological, and can be used to improve the quality of food service, production; targeting specific pathogens and their sources. Such data would allow more accurate estimates of the true burden of foodborne disease at national and regional levels. It has also been suggested that physicians may also be less likely to submit fecal samples for testing if the clinical signs demonstrated by the patient included respiratory signs [27] which does not automatically rule out gastrointestinal illness.

It is essential in acting in the spirit of preventive medicine, to increase the health literacy of the various stakeholders including health practitioners, which ultimately will have a positive impact on the morbidity and mortality associated with foodborne diseases [28]. By raising the awareness and understanding of the public on issues of food safety and foodborne diseases, the average consumer will be empowered to make better food consumption choices and also demand a higher level of service from vendors. A study by Badries et al. [29] focused on food vendors in Trinidad and Tobago, recognized that the vendors judged the doneness of hamburger patties by the color but not internal temperature, which exposes consumers to possibly undercooked meats. Many of the governments in the region have offered training and licensing programs to food vendors, however as the selling of food is often seen as an easy avenue to economic entrepreneurship in the region many of the food vendors are unlicensed and untrained, often due to the lack of resources or political will to enforce these policies. Webb and Morancie [30] found that the overall knowledge of food handlers in a survey of a university campus was poor even with formal training. This campaign should be also geared towards familiarizing the public about risky food hygiene practices at home and the risk associated with the culture of purchasing street-foods [31]. Most of the public does not understand the epidemiology of foodborne illness, often linking symptoms to meals consumed in only the last 24 h and indeed only drawing that link to the direct evidence of gastrointestinal signs of illness. Even when possible cases are reported it has been recognized that patients are often reluctant to submit samples for testing because of the strong distaste at having to collect samples [32]. 

## 4. Direct Action Needed

There is a significant paucity of information on health and the burden of disease in the Caribbean, especially related to foodborne disease. Today greater value is placed on evidence based medicine, and information on the disease burden of foodborne pathogens can be used to foster change in the attitudes of health practitioners, agriculturalists, food manufacturers, policy makers and the public on issues of food safety and disease. It is therefore incumbent upon the institutions within the region to engage in research, especially translational research that would be directly applicable to the challenges we face in the Caribbean. Not enough work is centered on the epidemiology of the microbial and parasitic food pathogens that impact the region, nor is there enough focus on Caribbean food chains and understanding ways to improve food safety while encouraging increased production in an attempt to meet the region’s food security challenges [33].

Some of this much needed information includes source attribution; identifying the foodstuffs most often associated with the pathogens common to the individual Caribbean territories and the region. The Caribbean has a tremendous food import bill of over $US4 billion which is projected to more than double by 2020 to $US8–10 billion [34] and many regional governments are calling for increased local production. Identifying food sources, especially locally produced foods, associated with pathogens of foodborne disease will provide data that can be used to improve these products and thus bolster our regional food production industries while maintaining food safety. Pires et al. [35] indicated that over the decades the major sources of foodborne illness have transitioned from mainly products of animal origin to crop products. This information should be used to direct our regional efforts to control foodborne illness, working with the farming, manufacturing and retail sectors as well as the final consumers. Many outbreaks of foodborne disease have driven home the fact that the quality and safety of the product available to the final consumer is dictated by the safety of inputs at every level of the food supply chain from the “farm to the fork” [36,37]. This likewise underscores the importance of source attribution and the early stage for preventive action for food safety.

Increased education of local farmers and food producers will be essential to supporting the development of national and regional food industries. Educating farmers and other producers on the roles they play regardless of the point in the food supply chain that they function; and providing upstream actions that they can take to improve food safety downstream for the final consumer will have exponential benefits. There are many initiatives targeting the education of producers across the region, many at the local level as with the former Consolidated Foods Limited in Saint Lucia which has been since sold. While these are a great first step, making these sort of programs mandatory as part of a licensing initiative for the various nodes of the food supply chain will be an additional step in the pathway to safer food.

Recommending the strengthening of national and regional surveillance is not a novel suggestion and many attempts have been made to address this challenge, recognizing its benefits will come at some significant financial cost [38]. The regional institutions such as the University of the West Indies (UWI) and agencies such as the CARPHA are critical to the collection, analysis and dissemination of the resulting information relevant to foodborne disease. Integrating information from animal health sources, agricultural data sources, food supply chain data and traditional health data is a desirable goal but requires careful strategic planning amidst the resource constraints of the Caribbean region. The implementation of electronic health record systems in at least some of the hospitals across the region is an important tool that can be used to build a real-time data analysis algorithm providing public health officers information that can be applied to the prevention and control of not only foodborne but other infectious and non-communicable diseases. This real-time surveillance should include data streams from the private sector anywhere along the food supply chain, as well as regional and international health data sources. 

The sameness of the experience of around foodborne diseases in the individual Caribbean territories is a strength not a weakness, recognizing that outbreaks may span multiple national borders similar to other infectious diseases and is an opportunity to collaborate and share public health resources. Though many of the desired improvements to food safety may call for the use of capital intensive technology and infrastructure, such as enhanced molecular diagnostic techniques, syndromic surveillance systems and the electronic networks to support such surveillance, it is important that the governments of the Caribbean come together with the other stakeholders to design a long-term strategy to improvement foodborne disease surveillance and food safety, which will include short and medium term goals. It is equally important that the long-term strategy must be realistic taking into consideration the capital and operational costs of the system and where these funds will be sourced. It does no good to design a state-of-the-art surveillance system, that while funding is available to design, build, and implement there is not regional commitment to fund the associated consumables, salaries and costs. The system must consider the difference in national priorities, needs and resources across the Caribbean while being harmonious and scalable. The short and medium term goals should include the identification of multidisciplinary surveillance goals with the input of a broad cross-section of stakeholders; drafting and enactment of the appropriate laws which will allow easier, secure transnational sharing of data; the systems architecture design that is the necessary precursor to a syndromic surveillance system [39] and by far the most important preventive medicine tool available to a public health practitioner; educational programs. Key to developing a strong surveillance system across the Caribbean is the utilization of the already trained personnel and specifically training other to address the gaps identified in the design stages of the surveillance plan, though in the Caribbean the lack laboratory reagents and other consumable resources are often the restricting factor. A strong corollary to improved and increased surveillance is the strengthening of response capacity across the region; centering on communication teams, public health practitioners and the supporting resources and infrastructure.

## 5. Conclusions

Both the regional tourism and agricultural industries could be decimated by an unfortunate incident of a foodborne disease epidemic. In 2008, the US saw tremendous loss to its tomato industry in the wake of a *Salmonella saintpaul* outbreak that was mistakenly linked to tomatoes; the State of Georgia lost almost $US14 million during the outbreak [40]. Analysis of the US stock market has shown that there is a decrease in the performance of companies linked to foodborne disease outbreaks [41] and single food recall can cost a company millions of dollars [42], in the Caribbean this could have disastrous outcomes to a struggling food industry with little market resiliency. Without the necessary data and subsequent analysis we continue to underestimate the true impact of foodborne diseases in the Caribbean. There are many avenues available to the countries of the Caribbean to address this challenge from educational campaigns to integrated surveillance to trans-national collaboration. This is an opportunity to institute a true One Health approach [43] to the challenge of food safety and food security [44] as well as food defense which is seldom if ever addressed in a Caribbean context. This would provide a broader platform to successfully address the “wicked problem” [18,45,46] of food safety and its underestimated impacts across the region.
